# A systematic review of triage-related interventions to improve patient flow in emergency departments

**DOI:** 10.1186/1757-7241-19-43

**Published:** 2011-07-19

**Authors:** Sven Oredsson, Håkan Jonsson, Jon Rognes, Lars Lind, Katarina E Göransson, Anna Ehrenberg, Kjell Asplund, Maaret Castrén, Nasim Farrohknia

**Affiliations:** 1 Department of Emergency Medicine, Helsingborg Hospital, Helsingborg, Sweden; 2 Department of Orthopedics, Uppsala University Hospital, Uppsala, Sweden; 3 Department of Management and Organisation, Stockholm School of Economics, Stockholm, Sweden; 4 Department of Medicine, Uppsala University Hospital, Uppsala, Sweden; 5 Department of Emergency Medicine, Karolinska University Hospital, Solna, Sweden; 6 Department of Medicine, Karolinska Institutet, Solna, Sweden; 7 School of Health and Social Studies, Dalarna University, Falun, Sweden; 8 Department of Public Health and Clinical Medicine, University Hospital, Umeå, Sweden; 9 Department of Clinical Science and Education and Section of Emergency Medicine, Södersjukhuset (Stockholm South General Hospital), Stockholm, Sweden; 10 Department of Emergency Medicine, Uppsala University Hospital, Uppsala, Sweden

## Abstract

**Background:**

Overcrowding in emergency departments is a worldwide problem. A systematic literature review was undertaken to scientifically explore which interventions improve patient flow in emergency departments.

**Methods:**

A systematic literature search for flow processes in emergency departments was followed by assessment of relevance and methodological quality of each individual study fulfilling the inclusion criteria. Studies were excluded if they did not present data on waiting time, length of stay, patients leaving the emergency department without being seen or other flow parameters based on a nonselected material of patients. Only studies with a control group, either in a randomized controlled trial or in an observational study with historical controls, were included. For each intervention, the level of scientific evidence was rated according to the GRADE system, launched by a WHO-supported working group.

**Results:**

The interventions were grouped into streaming, fast track, team triage, point-of-care testing (performing laboratory analysis in the emergency department), and nurse-requested x-ray. Thirty-three studies, including over 800,000 patients in total, were included. Scientific evidence on the effect of fast track on waiting time, length of stay, and left without being seen was moderately strong. The effect of team triage on left without being seen was relatively strong, but the evidence for all other interventions was limited or insufficient.

**Conclusions:**

Introducing fast track for patients with less severe symptoms results in shorter waiting time, shorter length of stay, and fewer patients leaving without being seen. Team triage, with a physician in the team, will probably result in shorter waiting time and shorter length of stay and most likely in fewer patients leaving without being seen. There is only limited scientific evidence that streaming of patients into different tracks, performing laboratory analysis in the emergency department or having nurses to request certain x-rays results in shorter waiting time and length of stay.

## Background

Overcrowding in emergency departments (EDs) is an increasing global problem [1]. In the United States, an Institute of Medicine committee has characterized ED overcrowding as a national crisis [2]. Emergency department overcrowding also compromises patient safety and timeliness (time to appropriate treatment) [3], threatens patient privacy and confidentiality, and often leads to frustration among ED staff [4-12].

Multiple factors determine patient flow in EDs, [13,14] and the *input-throughput-output conceptual model *has become an accepted approach toward understanding the causes of overcrowding [3,15,16]. According to the model, the causes may be sought in any of the three domains and actions to reduce overcrowding may be directed towards input, throughput or output from the ED. Although some of the suggested solutions to improve patient flow in EDs have arisen from systematic analyses, many improvements are of an ad hoc character [17]. Many of the new strategies are inspired by lean healthcare thinking with a focus on flow orientation, reduction in unnecessary work elements, continuous quality improvement, and participation of all co-workers [18-20].

Despite many efforts, scientific knowledge remains limited as regards which strategies and pragmatic approaches actually improve patient flow in EDs. The American Academy of Emergency Medicine recently released a statement concluding "it is currently unknown which strategies provide the best solution to fix throughput in the ED" [1].

In recent years, health authorities in many countries have introduced standards, with or without economic incentives, to decrease the length of stay in EDs [21]. The most well known is the 4-hour target set by the National Health Service (NHS) in the UK [22].

The objective of this review is to identify and evaluate the scientific evidence of various interventions to improve patient flow in EDs.

In 2010 The Swedish Council for Health Technology Assessment (SBU), a governmental agency, presented a systematic literature review to explore the scientific basis for various interventions to improve patient flow in EDs. The present review is based on data from this report [23].

## Methods

A systematic search of the international literature published from 1966 through March 31, 2009 was performed in British Nursing Index, Business Source Premier, CINAHL, Cochrane Library, EMBASE, ProQuest ABI, PubMed, and Science Direct (for search strategies see additional file 1). The database search was complemented by a thorough review of reference lists and review articles. Inclusion of papers was limited to studies of adult patients (≥15 years of age) visiting EDs for somatic reasons.

To be included, studies had to present data on waiting time (WT), i.e. the time interval between arrival at the ED and examination by a physician, length of stay (LOS), i.e. the total time spent in the ED, left without being seen (LWBS), i.e. the proportion of patients leaving the ED without being seen by a physician, or other flow parameters based on a nonselected material of patients. Studies were included only if they had a control group, either in a randomized controlled trial, or in an observational study with historical controls.

All studies were reviewed for quality by using validated checklists for internal validity, precision, and applicability (external validity) [24,25]. Methodological quality and clinical relevance of each study was graded as high, medium, or low. Two independent experts performed the review in a blinded manner and studies were only included if both experts considered the study as relevant. To reduce variation between the experts, standardized forms were used.

The second step involved using the internationally developed GRADE system to achieve an overall appraisal of the scientific evidence upon which the report's conclusions are based [26]. The following factors were considered when appraising the overall strength of evidence: study quality, concordance/consistency, transferability/relevance, precision of data, risk of publication bias, effect size, and dose-response. Predefined guidelines for up- and downgrading were used to arrive at the final grade indicating the strength of evidence [26]. Downgrading reflected limitations in study design or implementation, imprecision of estimates, variability in results, indirectness of evidence, or publication bias. Upgrading reflected a large magnitude of effect, a dose-response gradient, and consistency of data. Based on these rules, each conclusion was rated as having strong, moderately strong, limited, or insufficient scientific evidence. In the grading process, studies having low quality and relevance were included when studies of medium quality and relevance were not available.

## Results

### Literature search, selection process, and outcome measures

**Figure 1:**
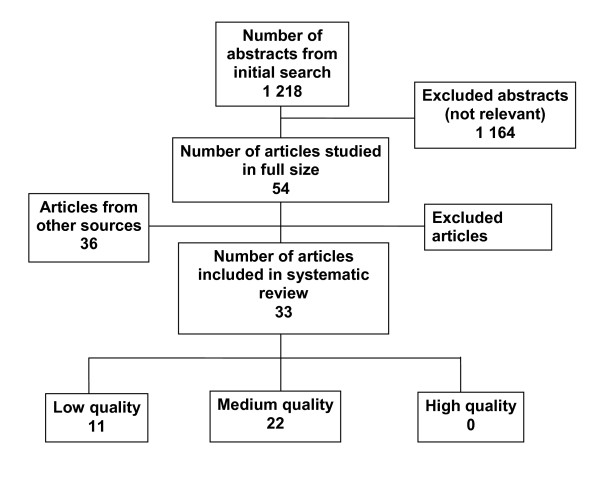
**Results of literature search and selection process**. (See separate file).

The initial search identified 1,218 abstracts, which were evaluated for relevance. Fifty-four articles were considered as potentially relevant and evaluated in full text. In addition, 36 articles were found by "snowballing", i.e. through reference lists and other sources. Ultimately, 33 articles were selected. The final selection was based on relevance, eligibility, and study design (Figure 1). Of these articles, none fulfilled the criteria for high quality, 22 were of medium quality, and 11 were of low quality.

The two most common outcome measures were WT (16 studies) and LOS (23 studies). Less common (11 studies) were reports on LWBS. Notably, none of the studies reported on indicators of patient safety or cost benefit.

The articles that were finally selected were divided into five groups, each group representing a specific type of intervention used to improve patient flow in the ED. The interventions are: streaming, fast track, team triage, point-of-care testing, and nurse-requested x-ray.

### Streaming

Streaming refers to routines where patients, following triage or brief evaluation, are divided into different processes (streams) according to more or less defined criteria. The most common example of streaming involves the use of a separate process, usually called fast track, to handle patients with less serious symptoms. Of the 16 studies on streaming that fulfilled the inclusion criteria [27-42], 13 focused on fast track and are reported separately (see below). The three remaining studies were of medium quality. Two of these studies separated patients into two processes (streams); patients who would benefit from admission and those who could be treated as outpatients [40,41]. The cohorts were large; 63,000 and 99,000 patients, respectively. King et al were unable to demonstrate shorter WT. However, LOS in the ED was reduced in both streams. Kelly et al reported reduced WT and shorter LOS for patients in 2 of 5 triage levels. The ED was also able to fulfil a 4-hour goal of WT to a greater extent with than without streaming. The third study divided patients of all categories into two streams where patients were cared for by two teams of physicians and nurses [42]. The method was called "team assignment" and reduced WT by 9 minutes on average, and the number of patients that left without being seen was reduced from 2.3% to 1.6% (Additional file 2).

Based on these studies, the scientific evidence for streaming, not including fast track, is limited (Table 1).

**Table 1:** Evaluation of scientific evidence of streaming according to GRADE.

Outcome measures	Number of patients (number of studies)	Study design	Outcome*, median (min-max)	Scientific evidence according to GRADE	Comments
Waiting time (shorter)	240 429 (3 studies)	Observational studies	31 (14-48) min	Limited ⊕⊕	Upgraded because of study quality. Downgraded because of outcome size
Length of stay (shorter)	141 017 (2 studies)	Observational studies	9.5 (0-11) min	Limited ⊕⊕	Downgraded because of study quality. Upgraded because of outcome size.

* Outcome calculated as the difference between intervention and control

### Fast track

Thirteen studies described the effects of fast track on patient flow in the ED [27-39]. Two of the studies were quasi-randomized, whereas the rest were prospective studies with historical (retrospective) control groups. Of the 13 studies, 9 were of medium quality and 4 were of low quality (Additional file 3).

Kilic et al published a quasi-randomized study where fast track was used every second day during daytime for 1 month [34]. During days without fast track, suitable patients were registered and used as controls. The study was relatively small with 143 patients in the study group and 126 patients in the control group. WT was significantly reduced with fast track.

A study in New Zealand evaluated and treated patients with less complicated problems via a separate process named the *Rapid Assessment Clinic *(RAC) during odd weeks [33]. Sixteen percent of all patients were selected to RAC. WT and LOS were reduced for patients in triage levels 4 and 5. The study indicated no effect on patients in the other triage levels.

In 2008, an Australian cohort study with 20,000 patients in each group (with or without fast track) demonstrated significantly shorter WT with fast track [30].

Another Australian study selected 33% of all patients to be treated by a senior physician in a fast track model [38]. WT was reduced, and the number of patients that left the ED without being seen dropped by 50%.

In a third study from Australia, O'Brien et al demonstrated reduced WT by 20% and reduced LOS by 18% for nonadmitted, fast track patients [35]. For patients that were eventually admitted, WT and LOS in the ED remained unchanged.

The largest study, an observational study originating from Spain, compared 71,000 fast track patients with an equally large control group [36]. Despite a 4.4% increase in attendance during the fast track period, WT was 50% shorter and LOS 10% shorter for the total patient population, when fast track was introduced. In this study, physician assistants and nurse practitioners staffed the fast track.

Another seven smaller studies also demonstrated significant effects of fast track (Additionel file 3).

In conclusion, all 13 studies demonstrated positive effects on WT and LOS when fast track was implemented. Based on these studies, the scientific evidence for improved patient flow following the implementation of fast track is moderately strong (Table 2).

**Table 2:** Evaluation of scientific evidence of fast track according to GRADE.

Outcome measures	Number of patients (number of studies)	Study design	Outcome*, median (min-max)	Scientific evidence according to GRADE	Comments
Waiting time (shorter)	>90 000 (9 studies)	1 RCT 8 observational studies	24.5 (2-51) min	Moderately strong ⊕⊕⊕	Upgraded because of outcome size and concordance of data
Length of stay (shorter)	>100 000 (10 studies)	2 RCT 8 observational studies	27 (4-74) min	Moderately strong ⊕⊕⊕	Upgraded because of outcome size and concordance of data
Number of patients leaving ED without being seen by a physician (fewer)	>90 000 (5 studies)	No RCT 5 observational studies	3.1 (0.2-4.1) percent	Moderately strong ⊕⊕⊕	Upgraded because of outcome size and concordance of data
Patient satisfaction (increased)	447 (2 studies)	1 RCT 1 observational study	-	Insufficient ⊕	Downgraded because of study quality, imprecise data and low reproducibility

* Outcome calculated as the difference between intervention and control for all patients or for patients leaving the ED if data is missing for all patients. If results only are presented per triage group calculations are made for triage group 4.

### Team triage

Team triage is defined as triage handled by a team that includes a physician. The rationale for team triage is to increase accuracy and efficiency in the initial process of patient evaluation. Six articles on team triage were included and reviewed, of which three were of medium quality and three were of low quality (Additional file 4) [43-48]. Two studies were quasi-randomized, and the remainders were prospective observational studies with retrospective controls.

A quasi-randomized study from Canada with 6,000 patients evaluated the effect of a triage liaison physician on LOS and LWBS [43]. The liaison physician facilitated patient flow by supporting the triage nurse, evaluating ambulance patients, initiating the diagnostic procedure, and handling administrative questions. Total LOS was reduced by 11% and LWBS was reduced by 20%.

In a study from Northern Ireland, Subash et al randomized approximately 1,000 patients to team triage or ordinary triage [44]. WT to see a physician was statistically reduced, as was the waiting time to x-ray. However, no reduction in total LOS could be demonstrated.

In a study from the United States, Partovi et al investigated the effect of a senior emergency physician in the triage team and reported that total LOS decreased by 82 minutes on average [47]. Using multivariate analysis, they showed that the effect was mainly the result of team triage, whether or not the patient was admitted, and whether or not x-ray was needed.

An Australian study with over 10,000 patients evaluated the effect of a Rapid Assessment Team (RAT) consisting of a physician and a registered nurse [48]. The WT targets were achieved in 59% with RAT, compared to 39% without RAT.

Based on the reviewed studies, we conclude that limited evidence suggests an effect of team triage on patient flow as measured by WT and LOS. However, relatively strong evidence suggests that team triage reduces the number of patients leaving the ED without being seen by a physician (Table 3).

**Table 3:** Evaluation of scientific evidence of team triage according to GRADE.

Outcome measures	Number of patients (number of studies)	Study design	Outcome*, median (min-max)	Scientific evidence according to GRADE	Comments
Number of patients leaving ED without being seen by a physician (fewer)	32 830 (4 studies)	1 RCT 3 observational studies	1.3 (1.2-6.8) percent	Moderately strong ⊕⊕⊕	Upgraded because of concordance of data
Waiting time (shorter)	25 927 (3 studies)	No RCT 3 observational studies	18 (16-20) min	Limited ⊕⊕	Downgraded because of study quality and heterogeneity
Length of stay (shorter)	29 674 (4 studies)	2 RCT 2 observational studies	40.5 (0-55) min	Limited ⊕⊕	Upgraded because of outcome size. Downgraded because of study quality.

* Outcome calculated as the difference between intervention and control

### Point-of-care testing

Point-of-care testing (POCT), which in this review refers to moving laboratory analysis to the ED, has been introduced by some hospitals to increase the speed of diagnosis in the ED. Six studies of POCT fulfilled the inclusion criteria [49-54]. Four of these studies were classified as medium quality and two as low quality (Additional file 5).

A randomized study from Canada demonstrated shorter LOS when laboratory analyses were performed at the ED, especially for nonadmitted patients [50]. However, the study was small and therefore had low statistical power. Another randomized study with 800 patients demonstrated significant changes in management, but no effect on LOS or admission rates [49].

In a US study, Lee-Lewandrowski et al found shorter turnaround time (i.e. the time from ordering laboratory tests to the results being available for the attending physician) and shorter LOS with POCT [51]. The study demonstrated high satisfaction among the staff.

The selection of laboratory tests available as POCT has a substantial impact on the results. In a US study by Parvin et al, almost 95% of the patients also needed central laboratory analyses to complement POCT. Consequently, POCT had no effect on the patients' length of stay [52].

Based on the studies assessed, the effect of POCT on turnaround time is supported by relatively strong evidence, whereas its effect on LOS is supported by only limited evidence (Table 4).

**Table 4:** Evaluation of scientific evidence of point of care testing according to GRADE.

Outcome measures	Number of patients (number of studies)	Study design	Outcome*, median (min-max)	Scientific evidence according to GRADE	Comments
Response time (shorter)	12 273 (3 studies)	No RCT 3 observational studies	51 (51-51) min	Moderately strong ⊕⊕⊕	Downgraded because of study quality. Upgraded because of outcome size.
Length of stay (shorter)	18 401 (5 studies)	2 RCT 3 observational studies	21 (-8-54) min	Limited ⊕⊕	Downgraded because of low reproducibility and heterogeneity

* Outcome calculated as the difference between intervention and control

### Nurse-requested x-ray

X-ray examination is another time-consuming process in the ED. To shorten waiting time, some hospitals have piloted a routine of nurse-requested x-ray. Of the three studies on nurse-requested x-ray that were included in this review, two were of medium quality and one was of low quality. All studies were randomized, in one case quasi-randomized (Additional file 6) [55-57].

In a British study including 1,800 patients, registered nurses could request x-ray examinations of injuries below the elbow and knee [57]. No specific training was given the nurses, and patients were separated by a triage nurse to *nurse first *or *doctor first*. In the group seen first by a nurse, LOS was reduced for patients that did not need an x-ray, whereas no difference was observed in the group needing an x-ray. Nurses ordered slightly more x-rays (4%) than physicians did.

In a study by Lindley-Jones et al, also performed in the UK, a triage nurse randomized orthopedic patients with suspected fracture to nurse request or doctor or nurse practitioner request [55]. Time to diagnosis was significantly shorter in the nurse request group. However, nearly 8% of patients that did not receive a nurse-requested x-ray did receive an x-ray following the physician's examination.

In a quasi-randomized study from Australia, a triage nurse requested x-rays on odd dates and a physician made the request on even dates [56]. The study included only patients with wrist or ankle injuries. The study reported no difference in LOS in the ED between the groups.

Based on these studies, the scientific evidence for shorter WT and/or LOS following nurse-requested x-ray was graded as limited (Table 5).

**Table 5:** Evaluation of scientific evidence of nurse-requested x-ray according to GRADE.

Outcome measures	Number of patients (number of studies)	Study design	Outcome*, median (min-max)	Scientific evidence according to GRADE	Comments
Waiting time and/or length of stay (shorter)	2 682 3 studies	RCT	10 (6-37) min	Limited ⊕⊕	Downgraded because of study quality, low reproducibility and heterogeneity

* Outcome calculated as the difference between intervention and control. Because of low numbers, waiting time and length of stay have been grouped together.

## Discussion

Of the five interventions addressed in this review, fast track demonstrates the best scientific evidence. In addition to improving patient flow, fast track would likely have benefits related to economics and patient satisfaction. However, this requires further evaluation. Concerning ethics and patient safety, it is important to note that many studies clearly demonstrate that the introduction of fast track does not negatively affect treatment and waiting times of patients with more severe diseases and injuries. However, none of the studies in this review have evaluated patient safety outcome measures, e.g. mortality and need for treatment in an intensive care unit.

Fast track for patients with uncomplicated diseases and injuries was introduced and evaluated in EDs of many countries already in the 1990s [58]. The main intention of fast track was to reduce the total number of patients staying in the ED, and thereby improve patient satisfaction and patient safety. Patients were usually selected for fast track based on the triage nurse's decision of appropriateness. Many hospitals have developed their own rules and inclusion criteria for fast track, e.g. superficial wounds, less severe allergic reactions, fractures and distortions of small joints and bones, dog and cat bites, and minor burns [25,34,37]. The proportion of patients suitable for fast track varies between 10% and 30% of total patients seen in the ED [27,33,35]. For practical reasons, fast track is usually in operation during peak hours, i.e. not during nights.

Some studies have serious limitations resulting from wide variations in staffing and patient selection. However, when triage levels and selection routines are clearly specified, the strength of the data in many studies is satisfactory.

In many countries, it has become increasingly common to refer patients with uncomplicated problems to primary care facilities outside the hospital [59-61]. Although such an approach can be tempting as an alternative to fast track, it raises warning signals about patient safety and patient satisfaction [62].

Some authors stress the importance of using a senior physician to staff the fast track [38]. Other studies, however, demonstrate positive effects when junior doctors [27] are engaged and when nurse practitioners manage fast track [31]. Hence, it is likely that the concept rather than the seniority of staffing plays a decisive role. Many authors emphasize correct patient selection [28,34,37]. Patients selected for fast track should be able to manage without too many diagnostic procedures, e.g. laboratory tests and x-rays. Another important factor involves directing fast track patients to specific areas in the ED, separate from areas where patients with higher medical priorities are managed.

Streaming of patients on the basis of presumed hospital admission did not appear to improve patient flow. Reduced WT and LOS were detected only among patients that could be discharged, which is in line with the positive results of fast track. Few relevant studies have been published on streaming other than fast track, limiting the chances of detecting strong evidence.

In Sweden, there has been a recent development of triage systems that combine streaming into different processes with refined triage scales based on vital signs and precise symptoms [63,64]. The rationale for these new systems of *process triage *has been to improve patient flow and to increase patient safety, but this has yet to be verified in published studies.

Although team triage has not been universally defined, it usually means that a team consisting of a physician and a nurse initially evaluates the patient. In some instances a receptionist or a nurse assistant complements the team. Team duties vary. To avoid "bottle necks" it is important that the total handling time per patient is short, which indirectly defines the tasks of the team. With a physician present in the team, it has become increasingly common to add procedures, e.g. ordering laboratory tests and x-rays. In some studies, patients with minor complaints receive final treatment from the team, similar to the principle of fast track. Most authors agree that the team should focus on initiating and planning patient treatment, whereas final treatment should be referred to the ordinary staff. The advantage of team triage may be most significant in complex situations, whereas noncomplex patients are better handled by fast track. Most authors emphasize the importance of a senior physician in team triage [44,45]. Working as a team also offers educational and training opportunities for inexperienced staff [46].

The main effect of team triage appears to be that fewer patients leave the ED without being seen by a physician. Such an effect is not surprising given the presence of a physician in the triage team. However, it is also an indirect effect of handling patients more rapidly, which in turn could benefit patient safety.

More than two thirds of all patients seeking help at an ED require laboratory tests [14]. The process of laboratory testing is usually complex and includes different steps, e.g. ordering, sampling, marking, transportation, analysis, reporting of results, interpretation, and informing the patient. A Belgian study reports that the process adds approximately 80 minutes to the LOS in the ED [65]. Several interventions have been applied to shorten the process of laboratory testing, e.g. early ordering, predefined test panels based on symptoms and/or suspected diagnosis, limitations on tests that can be ordered from the ED, faster transportation to the laboratory, and faster reporting systems. Point-of-care-testing (POCT), which involves moving analytical instruments to the ED, has also been suggested.

Introducing POCT to the ED significantly decreases turnaround time for the laboratory analyses that can be performed as POCT. The effect on WT and LOS depends on the range of tests that can be analyzed. As a consequence of technical advancements, the range of tests continues to expand, and thus the positive effect on LOS can be expected to increase in the future. In this process, it is essential to consider and evaluate the precision and reliability of the methods [66]. Low precision will affect patient safety and hamper the effects on flow - at least in the long-term.

X-ray examination is another time-consuming process in the ED. In many cases, it is evident at first presentation that the patient needs an x-ray. This has led to the routine of nurse-requested x-ray in many EDs. The routine is usually limited to x-ray of distal joints and bones in the hand, foot, wrist, and ankle [55-57].

One could expect that requesting x-ray examination early might reduce LOS. However, none of the included studies demonstrated such an effect. On the negative side of nurse-requested x-ray is the increased risk of needing additional x-rays following the physician's examination. This could probably be reduced by greater emphasis on education [55-57]. One of the studies [57] demonstrated shorter LOS for patients not needing x-ray, which again suggests that sorting out patients that require no further investigation has the greatest impact on patient flow [45].

There are some important limitations of this review. Some of the interventions influence the entire process, i.e. team triage, fast track, and other forms of streaming, while others affect only certain parts of the process, i.e. POCT and nurse-requested x-ray.

Fast track is the most studied intervention and the method supported by the strongest scientific evidence. However, it is reasonable to perceive additive, perhaps synergetic, effects between all of the interventions described in this review, and a broad approach is most likely the way to success. This is in line with lean thinking, comprising continuous improvement in all parts of the process [18,67]. As the process relies heavily on technology and human interaction, extensive staff involvement is essential.

Processes *in *the ED are interlaced and coherent with processes *before *and *after *the ED stay. Prehospital and primary care are examples of processes before, and the provision of hospital beds is an example of a process after the ED visit. Therefore, processes outside of the ED setting also need to be systematically reviewed and improved.

Finally, one must acknowledge the design limitations in many of the studies in this review. It is difficult to isolate the effect of an intervention when organizational issues interfere. Context-related factors and organizational placebo effects can play a stronger role than the intervention itself, often making it difficult to draw conclusions. The effects of different interventions are hard to isolate and depend on the local context. This calls for additional methodological approaches with sharper focus on underlying factors. Interventions may also have consequences on quality, patient and staff satisfaction, and economic and ethical issues, all of which must be taken into consideration. Consequently, further studies and new approaches are needed to fully evaluate the effects of organizational interventions.

## Conclusions

Introducing fast track for patients with less severe symptoms results in shorter waiting time, shorter length of stay, and fewer patients leaving without being seen. Team triage, with a physician in the team, will probably result in shorter waiting time and shorter length of stay and most likely in fewer patients leaving without being seen. There is only limited scientific evidence that streaming of patients into different tracks, performing laboratory analysis in the emergency department or having nurses to request certain x-rays results in shorter waiting time or length of stay.

## Competing interests

The authors declare that they have no competing interests.

## Authors' contributions

All authors participated in the design of the review. SO and HJ performed the analysis of the literature. All authors were part of conclusions and final grading. SO drafted the manuscript and all authors read and approved the final manuscript.

## Supplementary Material

Additional file 1 **Search strategies**. Click here for file

Additional file 2 **Streaming**. (Detailed analysis of reference [40-42]). Click here for file

Additional file 3 **Fast track**. (Detailed analysis of reference [27-39]. Click here for file

Additional file 4 **Team triage and similar interventions**. (Detailed analysis of reference [43-48]). Click here for file

Additional file 5 **Point-of-care testing**. (Detailed analysis of reference [49-54]). Click here for file

Additional file 6 **Nurse-requested x-ray**. (Detailed analysis of reference [55-57]). Click here for file
