# Supplementary material for: A systematic review of triage-related interventions to improve patient flow in emergency departments
Source: Scand J Trauma Resusc Emerg Med. 2011 Jul 19;19:43. doi: 10.1186/1757-7241-19-43 (PMC3152510; doi:10.1186/1757-7241-19-43)
Supplement: Additional file 1 — Search strategies. [file 1757-7241-19-43-S1.PDF]

Additional file 6.

Search strategies

PubMed
Patient flow (TiAb)
Patient turnover (TiAb)
Caseload (TiAb)
Case Load (TiAb)
Case loads (TiAb)
Caseloads (TiAb)
Workload (MeTiAb)
Work load (TiAb)
Workloads (TiAb)
Work loads (TiAb)
Department volume (TiAb)
Efficiency (Ti)
Effectiveness (Ti)
Waiting times (TiAb)
Length of stay (Me)
OR
Crowding (Me)
AND
Emergency (TiAb)
AND
Emergency service, hospital (Me)
Emergency nursing (Me)
Emergency departiment (TiAb)
Emergency departments(TiAb)

British Nursing Index (OVID HOST)
Patient flow (TiAb)
Patient turnover (TiAb)
Workload (TiAb)
Caseload (TiAb)
AND
Triage (TiAbDeHw)
Department volume (TiAb)
Clinic volume (TiAb)
Efficiency (Ti)
Effectiveness (Ti)

CINAHL (EBSCO HOST)
Workload (MH)
Organizational efficiency (MH+)
Patient flow* (TiAb)
Workload* (TiAb)

Caseload* (TiAb)
AND
Triage (SHMHTiAb)
Department volume* (TiAb)
Clinic volume* (TiAb)
Efficiency (Ti)
Effectiveness (Ti)

EMBASE
Workload (ExpTiAb)
Emergency health services (TiDe)
Patient flow (TiAb)
AND
Emergency nursing (TiDe)
Patient turnover (TiAb)
Emergency department (TiAb)
Workloads (TiAb)
Emergency departments (TiAb)
Work load (TiAb)
Emergency room (TiAb)
Work loads (TiAb)
Emergency ward (TiAbDe)
Caseload (TiAb)
Case load (TiAb)
Caseloads (TiAb)
Case loads (TiAb)
Department volume (TiAb)
Department volumes (TiAb)
Clinic volume (TiAb)
Clinic volumes (TiAb)
Efficiency (Ti)
Effectiveness (TiAb)
Waiting times (TiAb)
Length of stay (TiAbDe)
AND
Crowding (TiAbDe)
AND
Emergency (TiAb)

Cochrane Library
Patient flow (TiAb)
Emergency service, hospital (KW)
Patient turnover (TiAb)
AND
Emergency nursing (KW)
Waiting times (TiAb)
Emergency department (TiAb)
Workload (KWTiAb)

Emergency departmetns (TiAb)
Work load (TiAb)
Emergency ward (TiAb)
Caseload (TiAb)
Emergency wards (TiAb)
Caseloads (TiAb)
Case load (TiAb)
Department volume (TiAb)
Department volumes (TiAb)
Clinic volume (TiAb)
Efficiency (Ti)
Effectiveness (Ti)
Length of stay (KWTiAb)
OR
Crowding (TiAb)
AND
Emergency (TiAb)
