# Supplementary material for: A systematic review of triage-related interventions to improve patient flow in emergency departments
Source: Scand J Trauma Resusc Emerg Med. 2011 Jul 19;19:43. doi: 10.1186/1757-7241-19-43 (PMC3152510; doi:10.1186/1757-7241-19-43)
Supplement: Additional file 2 — Streaming. (Detailed analysis of reference [40-42]). [file 1757-7241-19-43-S2.PDF]

Additional file 2. Streaming

Author<br>Year, reference<br>Country<br>Journal	Study design	Size of emergency dept<br>Admission rate	Intervention (I)<br>Control (C)	Outcome	Results<br>Intervention (I)<br>Control (C)<br>Difference (D)	Study quality and relevance<br>Comments
Kelly AM et al<br>2007 [37]<br>Australia	Observational study<br>Prospective vs retrospective control<br>One year before and one year after intervention.	32 000/year<br>Admission rate 23%	I: Streaming into two processes (admission and discharge). Separate teams with senior emergency physician in each<br>N=31 500<br><br>C: No streaming and mixed patients<br>N=31 500	WT (NTS 3)<br><br>WT (NTS 5)<br><br>LOS (NTS 3)<br><br>LOS (NTS 4)<br><br>LOS (NTS 5)<br><br>Admitted within 4 hours<br><br>Discharged within 4 hours	I: 9 minutes<br>C: 14 minutes<br>D: 5 minutes<br>p<0.005<br><br>I: 45 minutes<br>C: 56 minutes<br>D: 11 minutes<br>p<0.005<br><br>I: 290 minutes<br>C: 283 minutes<br>D: - 7 minutes<br>p<0.02<br><br>I: 199 minutes<br>C: 213 minutes<br>D: 14 minutes<br>p<0.005<br><br>I: 115 minutes<br>C: 133 minutes<br>D: 18 minutes<br>p<0.005<br><br>I: 73%<br>C: 54%<br><br>I: 92%<br>C: 83%	Moderate<br><br>Shorter WT for NTS 3 and 5 with streaming.<br><br>Shorter LOS for NTS 4 and 5 with streaming<br><br>More patients to ward or discharged within 4 hours with streaming
King DL et al<br>2006 [38]<br>Australia	Observational study<br>Prospective vs retrospective control	50 000/year<br>Admission rate 43%	I: Streaming to discharge or admission<br>A- and B-team + resuscitation team<br>N=50 337	WT to see doctor (all)	I: 86 minutes<br>C: 86 minutes<br>D: 0<br>NS	Moderate<br><br>Shorter LOS for admitted as well as discharged patients but no increase in patients seen

	Twelve months before and 12 months after intervention.<br><br>All patients seen by triage nurse		C: No streaming<br>N=49 075	LOS (all)<br><br><br><br>LOS (admitted patients)<br><br><br>LOS (discharged patients)<br><br><br>Mortality<br><br><br><br>LWBS<br><br><br><br>LOS<4 hours	I: 5.0 hours<br>C: 5.8 hours<br>D: 0.8 hours (=48 min)<br>p<0.001<br><br>I: 7.0 hours<br>C: 8.5 hours<br>p<0.001<br><br>I: 3.4 hours<br>C: 3.7 hours<br>p<0.001<br><br>I: 0.11%<br>C: 0.10%<br>NS<br><br>I: 3.2%<br>C: 5.5%<br>p<0.001<br><br>I: 53%<br>C: 48%<br>p<0.001	within ATS threshold times with streaming
Patel PB et al 2005 [39]<br>USA	Observational study<br>Prospective vs retrospective control<br>One year before and one year after intervention.	39 000/year	I: Streaming to teams with 1 emergency physician, 2 nurses and 1 technician<br>Same patients to all teams<br>N=39 301<br><br>C: No streaming<br>N=38 716	WT<br><br><br><br><br><br>LWBS<br><br><br><br><br>Patient satisfaction	I: 61.8 minutes<br>C: 71.3 minutes<br>D: 9.5 minutes<br>95% CI = 5.8–13.5 minutes<br><br>I: 1.6%<br>C: 2.3%<br>Diff = 0.8 with 95% CI = 0.4–1.1%<br><br>I: Increase	Moderate<br><br>Shorter WT and fewer LWBS with streaming<br><br>Increased patient satisfaction<br><br>Very high numbers

WT = waiting time; LOS = length of stay; LWBS = left without being seen; NS = non significant; CI = confidence interval; ATS = Australasian Triage System; NTS = National Triage Scale
