# Supplementary material for: A systematic review of triage-related interventions to improve patient flow in emergency departments
Source: Scand J Trauma Resusc Emerg Med. 2011 Jul 19;19:43. doi: 10.1186/1757-7241-19-43 (PMC3152510; doi:10.1186/1757-7241-19-43)
Supplement: Additional file 3 — Fast track. (Detailed analysis of reference [27-39]. [file 1757-7241-19-43-S3.PDF]

Additional file 3. Fast track

Author<br>Year, reference<br>Country	Study design and included patients	Size of emergency dept<br>Admission rate	Intervention (I)<br>Control (C)	Outcome	Results<br>Intervention (I)<br>Control (C)<br>Difference (D)	Study quality and relevance<br>Comments
Rogers T et al<br>2004 [24]<br>United Kingdom	Observational study<br>Prospective vs. retrospective control (2–3 weeks before and after)<br><br>Triage category 4 (not specified)	59 000/year	I: FT 8 am–6 pm Monday to Friday w senior house officer and nurse practitioners<br><br>C: No FT	WT to see doctor or nurse practitioners<br><br>LOS<br><br>Discharge in 4 hours	I: 30 minutes<br>C: 56 minutes<br>D: 26 minutes<br><br>I: 1h 17 minutes<br>C: 1 h 39 minutes<br>D: 22 minutes<br><br>I: 92%<br>C: 87%	Low<br><br>Shorter WT and LOS<br><br>No statistics<br><br>No numbers
Fernandes CM et al<br>1996 [25]<br>Canada	Observational study<br>48 hours periods (before and after)	54 000/year	I: Changing of FT (larger area, full-time nurse)<br>N=106<br><br>C: FT without changes<br>N=100	LOS (only FT)<br><br>LOS (all patients)	I: 64 minutes<br>C: 82 minutes<br>D: 18 minutes<br>p<0.05<br><br>I: 114 minutes<br>C: 115 minutes<br>D: 1 minute<br>NS	Moderate<br><br>Shorter LOS for FT-patients without effects on other patients<br><br>Low numbers
Darraab AA et al<br>2006 [26]<br>Canada	Observational study<br>1 week of intervention vs same week in previous year<br><br>CTAS 3/4/5	38 000/year<br>Admission rate 18%	I: FT during 1 pm–7 pm all days<br>N=265<br><br>C: No FT<br>N=248	LOS (CTAS 4/5)<br><br>LOS (CTAS 3)<br><br>LWBS (CTAS 4/5)	I: 110 minutes<br>C: 170 minutes<br>D: 60 minutes<br>p=0.95<br><br>I: 60 minutes<br>C: 66 minutes<br>D: 6 minutes<br>p<0.001<br><br>I: 2%<br>C: 6%<br>D: 4 %<br>p=0.043	Moderate<br><br>Shorter LOS for CTAS 3<br>Lower LWBS for CTAS 4 and 5<br><br>Low numbers
Kwa P et al<br>2008 [27]<br>Australia	Observational study<br>6 months of intervention vs control	53 000/year<br>Admission rate 21%	I: FT (8 beds, 2 doctors, 2 nurses, open: 8 am–10 pm every day)	WT(% met target, ATS 4)	I: 79.9%<br>C: 77.8%<br>p<0.001	Moderate<br><br>Shorter WT for ATS 4

	(before and after)<br><br>ATS 4 for FT		N=20 460<br>(FT=3 047)<br><br>C: No FT<br>N=18 267	WT (ATS 4)<br><br><br><br><br>LOS (ATS 4)<br><br><br><br><br>LWBS	I: 22 minutes<br>C: 24 minutes<br>D: 2 minutes<br>p<0.001<br><br>I: 114 minutes<br>C: 110 minutes<br>D: 4 minutes<br>p=0.06<br><br>I: 3.3%<br>C: 3.5%<br>D: 0.2%<br>p=0.45	High numbers
Cooke MW et al<br>2002 [28]<br>UK	Observational study<br>Prospective vs retrospective control<br>5 weeks (before and after)<br><br>Patients with minor injury<br>without need of bed or intervention to FT (=triage category 4 and 5)	73 000/year	I: FT with junior doctor open 9 am–11 pm<br>N=6 801<br><br>C: No FT<br>N=7 117	WT to doctor<br><30 minutes<br><br><br><br><60 minutes<br><br><br>Within target<br>Triage category 2<br><br><br><br>Triage category 3<br><br><br><br>Triage category 4<br><br><br><br>Triage category 5	 I: 44%<br>C: 35.4%<br>p<0.0001<br><br>I: 76.2%<br>C: 65.1%<br>p<0.0001<br><br>I: 32%<br>C: 41%<br>NS<br><br>I: 78.6%<br>C: 72.8%<br>p<0.0001<br><br>I: 94.1%<br>C: 87.6%<br>p<0.0001<br><br>I: 100%<br>C: 96.1%<br>NS	Moderate<br><br>Only trauma<br><br>Shorter WT for triage category 3 and 4
Bond PA<br>2001 [29]<br>Saudi Arabia	Observational study<br>Retrospective analysis of 200 randomized charts<br>1 month before and	68 000/year	I: Physician and nurse staffed patient assessment room (PAR) for non urgent patients	WT	I: 25 minutes<br>C: 58 minutes<br>D: 33 minutes<br>p<0.05	Low<br><br>Shorter WT for non-urgent patients with PAR

	200 charts 1 month after<br><br>Non urgent patients to FT		N=200<br><br>C: No PAR<br>N=200			Low numbers
Ardagh MW et al 2002 [30]<br>New Zeeland	RCT<br>10 weeks: FT odd weeks and no FT even weeks.<br><br>All patients	65 000/year	I: Rapid assessment clinic (RAC) Monday to Friday 9 am–5 pm<br>N=2 263 with 361 to RAC<br><br>C: No RAC<br>N=2 204<br>of which 349 likely to RAC	WT to see doctor<br>ATC 2<br><br><br><br>ATC 3<br><br><br><br>ATC 4<br><br><br><br>ATC 5<br><br><br><br>LOS<br>ATC 2<br><br><br><br>ATC 3<br><br><br><br>ATC 4<br><br><br><br>ATC 5	 I: 8.2 minutes<br>C: 7.7 minutes<br>D: -0.5 minutes<br>NS<br>I: 29.7 minutes<br>C: 28.4 minutes<br>D: -1.3 minutes<br>NS<br><br>I: 34.5 minutes<br>C: 42.7 minutes<br>D: 8.2 minutes<br>p=0.004<br><br>I: 34.3 minutes<br>C: 45.4 minutes<br>D: 11 minutes<br>p=0.02<br><br>I: 172 minutes<br>C: 193 minutes<br>D: 21 minutes<br>NS<br><br>I: 190 minutes<br>C: 191 minutes<br>D: 1 minute<br>NS<br><br>I: 131<br>C: 158<br>D: 27 minutes<br>p=0.03<br><br>I: 65 minutes<br>C: 85 minutes<br>D: 20 minutes	Moderate<br><br>Shorter WT and LOS for ATC 4 and 5 with NS change for other patients

					p=0.06	
Kilic YA et al<br>1998 [31]<br>Turkey	RCT<br>analysis during 1 month, FT every other day<br><br>Patients included according to FT criteria<br>without life-threats	30 000/year	I: FT open 8 am–5.30 pm, Monday to Friday<br>N=143<br><br>C: No FT but registration of FT-cases<br>N=126	LOS of FT-patients<br><br><br><br>Patient satisfaction	I: 36 minutes<br>C: 63 minutes<br>D: 27 minutes<br>p<0.001<br><br>I: Improved	Moderate<br><br>Shorter LOS for patients in FT process<br><br>Low numbers
O'Brien D et al<br>2006 [32]<br>Australia	Observational study<br>12 weeks trial compared to same period previous year<br><br>ATS 3, 4 and 5 likely to be discharged (=21.6% of all patients)	43 000/year<br>Admission rate 48%	I: FT open 9 am–10 pm, Monday to Friday + 9.30 am–6 pm, Saturday and Sunday<br>Junior doctor + nurse<br>N=1 482<br><br>C: No FT<br>N=not specified	LOS of all discharged patients<br><br><br><br>WT of all discharged patients<br><br><br><br>LWBS	I: 186.5 minutes<br>C: 227.5 minutes<br>D: 41 minutes<br>Sign (95% CI)<br><br>I: 59.4 minutes<br>C: 74.4 minutes<br>D: 15 minutes<br>Sign (95% CI)<br><br>I: 1.5%<br>C: 2.2%<br>D: 0.7%<br>Sign (95% CI)	Low<br><br>LOS and WT shorter for discharged patients with FT<br><br><br>WT unchanged for admitted patients with FT
Sanchez M et al<br>2006 [33]<br>Spain	Observational study<br>one year of intervention vs one year before (control)<br><br>Non-urgent patients selected by triage nurse<br>(approx 30% of all patients)	75 000/year<br>Admission rate 21%	I: FT with physician assistant and nurse practitioners<br>open: 8.30 am–11 pm<br>N=71 000 (all pat)<br><br>C: No FT<br>N=75 000 (all pat)	WT (all patients)<br><br><br><br><br>LOS (all patients)<br><br><br><br><br><br>LWBS (all patients)<br><br><br><br><br>Mortality (all patients)	I: 51 minutes<br>C: 102 minutes<br>D: 51 minutes<br>p<0.001<br><br>I: 258 minutes<br>C: 286 minutes<br>D: 28 minutes<br>p<0.001<br><br><br>I: 3.72%<br>C: 7.78%<br>D: 4.06%<br>p<0.001<br><br>I: 0.27%<br>C: 0.28%	Moderate<br><br>Shorter WT and LOS for all patients with FT<br>Lower LWBS<br><br>No change in mortality and revisit rate

				Revisit rate (all patients)	NS<br><br>I: 4.51%<br>C: 4.57%<br>NS	
Rodi SW et al<br>2006 [34]<br>USA	Observational study<br>Prospective, retrospective control<br><br>CTAS 4+5	30 000/year	I: FT with physician assistant and emergency department technician<br>open: 9 am–7 pm<br>N=91<br><br>C: No FT<br>N=87	Patient satisfaction (excellent or very good)<br><br><br><br>LOS	I: 86%<br>C: 61%<br>p<0.001<br><br><br>I: 53 minutes<br>C: 127 minutes<br>D: 74 minutes<br>p<0.001	Low<br><br>Shorter LOS with FT<br><br>Increased patient satisfaction<br><br>Low number
Ieraci S et al<br>2008 [35]<br>Australia	Observational study<br>Prospective analysis of 6 months before and 6 months after<br>Patients not requiring a bed (approx 30% of all patients) to FT<br><br>All patients included in analysis	40 000/year	I: FT with senior doctor and nurse<br>16 hours/day<br><br>C: No FT	WT<br><br><br><br><br>Compliance w targets<br><br><br><br><br>LWBS<br><br><br><br><br>Revisit rate within 48 hours	I: 32 minutes<br>C: 55 minutes<br>D: 23 minutes<br>p<0.001<br><br>I: 77%<br>C: 60%<br>p<0.001<br><br>I: 3.1%<br>C: 6.2%<br>D: 3.1%<br>p<0.001<br><br>I: 4.0%<br>C: 3.2%<br>p<0.001	Moderate<br><br>Shorter WT for all patients with FT<br><br>Lower LWBS for all patients with FT<br><br>Small increase of revisit rate with FT
Considine J et al<br>2008 [36]<br>Australia	Observational study of matched case-control<br>Before/after<br><br>Non-urgent patients expected to be discharged and expected LOS <60 minutes to FT	70 000/year<br>Admission rate 25%	I: FT 10 am–2 am<br>Nurse, junior doctor or nurse practitioners<br>N=822<br><br>C: No FT<br>N=822<br>(matched in pairs)	WT<br>ATS 3<br><br><br><br><br>ATS 4	 I: 13 minutes<br>C: 12 minutes<br>D: -1 minute<br>NS<br><br>I: 29 minutes<br>C: 31 minutes<br>D: 2 minutes<br>NS	Moderate<br><br>Shorter LOS for discharged patients with FT<br><br>No change in WT for ATS 3–5 with FT

				ATS 5<br><br><br><br>LOS<br>Discharged patients<br><br><br><br><br>Admitted patients	I: 26 minutes<br>C: 25 minutes<br>D: - 1 minute<br>NS<br><br>I: 116 minutes<br>C: 132 minutes<br>D: 16 minutes<br>p<0.01<br><br>I: 309 minutes<br>C: 313 minutes<br>D: 4 minutes<br>NS	

FT = fast track; WT = waiting time; LOS = length of stay; LWBS = left without being seen; CTAS = Canadian Emergency Department Triage and Acuity Scale; ATS = Australasian Triage Scale; ATC = Australasian Triage Category
