# Supplementary material for: A systematic review of triage-related interventions to improve patient flow in emergency departments
Source: Scand J Trauma Resusc Emerg Med. 2011 Jul 19;19:43. doi: 10.1186/1757-7241-19-43 (PMC3152510; doi:10.1186/1757-7241-19-43)
Supplement: Additional file 4 — Team triage and similar interventions. (Detailed analysis of reference [43-48]). [file 1757-7241-19-43-S4.PDF]

Additional file 4. Team triage and similar interventions

**Author**<br>**Year, reference**<br>**Country**	**Study design**	**Size of emergency dept**<br>**Admission rate**	**Intervention (I)**<br>**Control (C)**	**Outcome**	**Results**<br>**Intervention (I)**<br>**Control (C)**<br>**Difference (D)**	**Study quality and relevance**<br>**Comments**
Holroyd BR et al<br>2007 [40]<br>Canada	RCT<br>Randomisation of shifts during 3 two-week periods<br>During each 2 week-period: 7 shifts (11 am–8 pm) with and 7 shifts without triage physician	55 000/year	I: Triage physician (initiate, assist triage, consult per telephone, discharge)<br>N=2 831<br><br>C: No triage physician<br>N=2 887	LOS<br><br><br><br><br>LWBS<br><br><br><br><br><br>Staff satisfaction	I: 4 hours 21 minutes<br>C: 4 hours 57 minutes<br>D: 36 minutes<br>$p<0.001$<br><br>I: 5.4%<br>C: 6.6%<br>D: 1.2%<br>$p<0.02$<br><br>80–90% positive	Moderate<br><br>Shorter LOS and fewer LWBS with triage physician<br><br>High staff satisfaction
Subash F et al<br>2004 [41]<br>Northern Ireland	RCT<br>Selection of 8 days during 4 consecutive weeks.<br>Randomisation of 4 shifts with and 4 shifts without team triage	50 000/year	I: Team triage 9 am–12 am (physician + nurse in triage)<br>N=530<br><br>C: No team triage<br>N=498	LOS (during 9 am–12 am)<br><br><br><br>Time to x-ray<br><br><br><br>Time to analgesia	I: 37 minutes<br>C: 82 minutes<br>D: 45 minutes<br>$p<0.057$<br><br>I: 11.5 minutes<br>C: 44 minutes<br>$p<0.029$<br><br>I: 13 minutes<br>C: 37.5 minutes<br>$p<0.4$	Low<br><br>Shorter LOS and time to x-ray with team triage
Travers JP et al<br>2006 [42]<br>Singapore	Observational study<br>Prospective w retrospective control.<br>10 days with team triage and 10 days without team triage<br><br>Only triage category 3	Size not described	I: Senior emergency physician in triage with nurse (10 am–4 pm)<br>N=290<br><br>C: No emergency physician in triage<br>N=286	WT to see doctor in treatment area (triage category 3)	I: 19 minutes<br>C: 35.5 minutes<br>D: 16.5 minutes<br>$p<0.05$	Low<br><br>Shorter WT with physician in triage<br><br>Low numbers
Richardsson JR et al<br>2004 [43]	Observational study<br>Prospective retrospective control.	39 000/year	I: Senior emergency physician in triage (to initiate treatment, order	WT to see doctor within thresholds<br>Triage category 3	I: 78%<br>C: 67%<br>$p<0.0001$	Low<br><br>Shorter WT with physician in

Australia	Three months before and 3 months after intervention		x-ray and lab and sometimes discharge)<br>N=2 193<br><br>C: No emergency physician in triage<br>N=1 991	Triage category 4<br><br>LWBS<br><br>Staff satisfaction	I: 73%<br>C: 53%<br>p<0.0001<br><br>I: 5.1%<br>C: 6.3%<br>D:1.2%<br>p<0.024<br><br>86% positive	triage
Partovi SN et al<br>2001 [44]<br>US	Prospective Observational study Eight Mondays 9 am to 9 pm with and 8 Mondays without team triage	52 000/year<br>Admission rate 16%	I: With additional senior physician in triage (to order diagnostic studies, fluid, discharge direct from triage)<br>N=920<br><br>C: Without senior physician in triage<br>N=841	LOS<br><br>LWBS	I: 363 minutes<br>C: 445 minutes<br>D: 82 minutes<br><br>Medel: -82 minutes (95% CI = -111 to -54 minutes)<br><br>I: 7.9%<br>C: 14.7%<br>D: 6.8%<br>p=0.068	Moderate<br><br>Shorter LOS with team triage<br><br>Fewer LWBS with team triage
Grant S et al<br>1999 [45]<br>Australia	Observational study. Prospective vs retrospective control 3 months before and 3 months after intervention	40 000/year	I: Rapid assessment team (physician and nurse). Initiating diagnostics and treatment.<br>N=10 691<br><br>C: Regular triage<br>N=10 476	WT to see doctor (median)<br><br>Seen in required time<br><br>LWBS (numbers (%))<br><br>LOS (median)	I: 32 minutes<br>C: 50 minutes<br>D: 20 minutes<br>p<0.001<br><br>I: 59%<br>C: 39%<br>p<0.001<br><br>I: 518 (4.9%)<br>C: 685 (6.4%)<br>D: 1,5%<br>NS<br><br>I: 3.2 hours<br>C: 3.2 hours<br>D: 0<br>NS	Moderate<br><br>Shorter WT with rapid assessment team<br><br>Fewer LWBS<br><br>Same LOS

LOS = length of stay; WT = waiting time; LWBS = left without being seen;
