# Supplementary material for: A systematic review of triage-related interventions to improve patient flow in emergency departments
Source: Scand J Trauma Resusc Emerg Med. 2011 Jul 19;19:43. doi: 10.1186/1757-7241-19-43 (PMC3152510; doi:10.1186/1757-7241-19-43)
Supplement: Additional file 5 — Point-of-care testing. (Detailed analysis of reference [49-54]). [file 1757-7241-19-43-S5.PDF]

Additional file 5. Point of care testing

**Author**<br>**Year, reference**<br>**Country**	**Study design**<br><br>**Patient population**	**Size of emergency dept**<br>**Admission rate**	**Intervention (I)**<br>**Control (C)**	**Outcome**	**Results**<br>**Intervention (I)**<br>**Control (C)**<br>**Difference (D)**	**Study quality and relevance**<br>**Comments**
Kendall J et al<br>1998 [46]<br>England	RCT<br>Random 8-hour periods during 1 year with and without POCT. Total of 210 periods<br><br>All patients	50 000/year	I: POCT<br>N=860<br><br>C: Central lab<br>N=868	Change in management<br><br>Mortality (in-hospital)<br><br>LOS<br><br>Admission rate	I: 6.9%<br>(earlier decision)<br>C: 5.3–8.8%<br>p<0.0001<br><br>I: 6.4%<br>C: 5.5%<br>p=0.45<br><br>I: 188 minutes<br>C: 193 minutes<br>D: 5 minutes<br>p=0.3<br><br>I: 85.2%<br>C: 83.5%<br>p=0.3	Moderate<br><br>Significant change in management with POCT but no change in mortality, LOS or admission rate
Murray RP et al<br>1999 [47]<br>Canada	RCT<br>During 5 months with inclusion of those suitable for only POCT-analysis (5% of all pat)	41 000/year	I: POCT<br>N=93<br><br>C: Central lab<br>N=87	LOS (all)<br><br>LOS (discharged)	I: 3 hours 28 minutes<br>C: 4 hours 22 minutes<br>D: 54 minutes<br>p<0.02<br><br>I: 3 hours 05 minutes<br>C: 4 hours 17 minutes<br>D: 72 minutes<br>p<0.001	Low<br><br>Shorter LOS for all patients with POCT<br><br>Low numbers
Lee-Lewandrowski E et al<br>2003 [48]<br>USA	Observational study<br>Prospective vs. retrospecitve control. Before and after intervention	70 000/year	I: POCT<br>(8 am–5 pm)<br>N=316<br><br>C: Central lab<br>N=271	TAT<br><br>LOS	I: 8 minutes<br>C: 59.5 minutes<br>D: 51 minutes<br>p=0.02<br><br>I: 347 minutes<br>C: 389 minutes<br>D: 42 minutes	Low<br><br>Shorter TAT, LOS and increased clinician satisfaction with POCT<br><br>Low numbers

				 <br>Clinician satisfaction	p<0.006<br><br>I: 4.3 (of max 5)<br>C: 1.95<br>p<0.001	
Parvin CA et al 1996 [49] USA	Observational study Prospective w three periods : control – intervention – control.	57 000/year	I: POCT (handheld) during 5 weeks<br>N=1 722<br><br>C: Central lab<br>Retro and prospective during 5+3 weeks<br>N=2 918	LOS	I: 209 minutes<br>C: 201 minutes<br>D: - 8 minutes<br>NS	Moderate<br><br>No change in LOS with POCT<br><br>95% of patients in intervention also needed central lab tests
Tsai WW et al 1994 [50] USA	Observational study Prospective analysis of 210 patients during 4 weeks (Monday to Friday) with split samples, one for POCT the other to central lab	Not described	I: POCT<br>N=210<br><br>C: Central lab<br>N=210<br>(same group as intervention group)	TAT<br><br><br><br>Possible earlier intervention	I: 8 minutes SD 6<br>C: 59 minutes SD 33<br>D: 51 minutes<br>No other statistics<br><br>I: 19%<br>C: –	Moderate<br><br>Shorter TAT and possible earlier intervention with POCT
Singer AJ et al 2008 [51] USA	Observational study Prospective w retrospective control One month before and one month after intervention	75 000/year Admission rate 20%	I: Specified lab for emergency dept analysis located at central lab<br>N=5 635<br><br>C: Regular central lab<br>N=5 631	% TAT within 30 minutes<br><br><br>LOS	I: 83–98%<br>C: 0.4–81%<br>p<0.001<br><br>I: 185 minutes<br>C: 206 minutes<br>D: 21 minutes<br>p<0.001	Moderate<br><br>Shorter TAT and LOS with POCT

POCT = point of care testing; TAT= Turn-around-time; LOS = length of stay
