# Supplementary material for: A systematic review of triage-related interventions to improve patient flow in emergency departments
Source: Scand J Trauma Resusc Emerg Med. 2011 Jul 19;19:43. doi: 10.1186/1757-7241-19-43 (PMC3152510; doi:10.1186/1757-7241-19-43)
Supplement: Additional file 6 — Nurse-requested x-ray. (Detailed analysis of reference [55-57]). [file 1757-7241-19-43-S6.PDF]

Additional file 6. Nurse-requested x-ray

**Author<br>Year, reference<br>Country**	**Study design**	**Size of emergency dept<br>Admission rate**	**Intervention (I)<br>Control (C)**	**Outcome**	**Results<br>Intervention (I)<br>Control (C)<br>Difference (D)**	**Study quality and relevance<br>Comments**
Lindley-Jones M et al<br>2000 [52]<br>United Kingdom	RCT<br>Two separate two week periods, six months apart.<br>Limb injuries except elbow, knee and hip	59 000/year	I: X-ray requested by triage nurse if needed (68%)<br>N=335<br><br>C: Regular triage and x-ray requested by nurse practitioners or emergency physician<br>N=340	WT (time to finishing assessing injury)	I: 65.5 minutes<br>C: 102.7 minutes<br>D: 37 minutes<br>p<0.0001	Moderate<br><br>Shorter WT for patients with nurse-requested x-ray<br><br>Nurses requested 8% fewer x-rays than doctors
Parris W et al<br>1997 [53]<br>Australia	RCT<br>Intervention on odd dates<br>Isolated injury to wrist or ankle<br>Patients that did not need x-ray or that were admitted were excluded	35 000/year	I: X-ray requested by triage nurse<br>N=87<br><br>C: X-ray requested by physician<br>N=87	LOS<br>No fracture N=121<br><br><br><br><br>Fracture N=55	 I: 100 minutes<br>C: 114 minutes<br>D: 14 minutes<br>p=0.14<br><br>I: 173 minutes<br>C: 179 minutes<br>D: 6 minutes<br>p=0.37	Low<br><br>No significant change in LOS if triage nurse initiated x-ray
Thurston et al<br>1996 [54]<br>United Kingdom	RCT, multicentre.<br>Triage nurse randomly allocated patients by random list to nurse or doctor.<br>Only limb injuries below elbow and knee	43 000–86 000/year<br>(4 hospitals)	I: X-ray requested by nurse<br>N=915<br><br>C: X-ray requested by doctor<br>N=918	LOS (all)<br><br><br><br><br>LOS (no x-ray)<br><br><br><br><br>Proportion of patients referred to x-ray	I: 88.5 minutes<br>C: 94 minutes<br>D: 6 minutes<br>p=0.1<br><br>I: 36 minutes<br>C: 51 minutes<br>D: 15 minutes<br>p<0.001<br><br>I: 78%<br>C: 74%<br>p=0.05	Moderate<br><br>167 patients excluded because of incomplete protocols or missing data<br><br>No difference in LOS except for patients where nurses did not request x-ray<br>More x-rays requested by nurses<br>Doctors added x-rays requests in 24% of nurse non required group

LOS = Length of stay; WT = Waiting time
